# Non-sputum-based triage and confirmatory diagnostic tests for pediatric TB

**DOI:** 10.5588/ijtldopen.24.0484

**Published:** 2025-03-12

**Authors:** A. Drane, A. Molkenthin, M. Gassama, S. Pouzol, P. Vanhems, J. Hoffmann

**Affiliations:** ^1^ George Washington University, Milken Institute School of Public Health, Washington, DC, USA;; ^2^ Mérieux Foundation USA, Washington, DC, USA;; ^3^ Fondation Mérieux, Lyon, France;; ^4^ PHE3ID Team, Centre International de Recherche en Infectiologie, Inserm U1111, CNRS UMR5308, ENS de Lyon, Lyon 1 University, Lyon, France.

**Keywords:** tuberculosis, pediatric TB, non-sputum diagnostics, triage, diagnostics

## Abstract

**BACKGROUND:**

Non-sputum-based triage and confirmatory tests are essential for early TB detection and timely treatment in children.

**METHODS:**

A mini-review was conducted from January 2022 to May 2024, evaluating five studies on non-sputum-based assays for childhood TB diagnosis. Both Microbiological and Clinical Reference Standards were used to assess diagnostic accuracy and triage potential.

**RESULTS:**

Among the confirmatory tests, only the gastric aspiration test with cartridge-based nucleic acid amplification tests (CBNAAT) met the WHO Target Product Profile criteria. However, this method remains invasive and is not suitable for point-of-care testing. Urine testing by gas chromatography–mass spectrometry (GC/MS) or C-ELISA (BJ76/A194) demonstrated high performance but lacked point-of-care applicability in resource-limited settings. Stool testing with CBNAAT is a viable alternative with high specificity but low sensitivity. For triage, urine lipoarabinomannan tests and blood MTB-HR tests show promise based on specificity, practicality, cost, and turnaround time.

**CONCLUSION:**

This review highlights the performance of non-sputum-based assays for childhood TB and their potential as triage tools. While some other innovations show promise for the triage and/or diagnosis of TB in adults, further studies are needed to evaluate the performance of these tests in pediatric populations.

TB remains a leading cause of death worldwide, with around 10 million new cases and 1.5 million deaths annually.^1^ Childhood TB, affecting those under 15, represents about 11% of global TB cases but is significantly under-diagnosed, with only about half of the cases reported to National Tuberculosis Programs (NTPs).^2,3^

The under-diagnosis of childhood TB stems from several factors, including the limited availability of robust non-sputum-based diagnostics. The paucibacillary nature of TB in children and difficulties in sputum collection hampers the effectiveness of traditional sputum-based tests such as culture, smear microscopy, and cartridge-based nucleic acid amplification tests (CBNAATs).^4,5^ Consequently, diagnosing childhood TB often relies on clinical assessments, radiological examinations (e.g., chest X-ray), immunological tests (e.g., tuberculin skin test [TST] and interferon-gamma release assays [IGRA]), and sometimes CBNAATs on sputum or stool. This composite approach is challenging in resource-poor settings and primary healthcare centres.

The WHO recommends using TB treatment decision algorithms (TDAs) to address the diagnostic gap, integrating microbiological, clinical, and radiological evidence.^2^ The TDAs advocate for molecular testing (CBNAAT) of respiratory or stool specimens and, where possible, urine samples for lipoarabinomannan (LAM) detection in children with HIV. Despite this, both methods often exhibit poor sensitivity, leading to missed TB cases in children. Effective non-sputum-based triage and confirmatory tests are essential for early detection and timely treatment of childhood TB.

Triage tests should be simple, low-cost, and suitable for first-contact providers, such as community health workers, to identify high-risk individuals who need further confirmatory testing rapidly.^6^ High specificity is crucial in triage tests to correctly identify those without TB, thus reducing false positives and unnecessary confirmatory testing. While high sensitivity is important for early detection and reducing transmission, particularly in rapidly progressing pediatric cases, it must be balanced with specificity to prevent overwhelming diagnostic resources.

Confirmatory diagnostic tests, on the other hand, should have high sensitivity to detect TB cases and guide treatment initiation accurately.^6,7^ These tests are typically more complex and costly, often requiring advanced technologies like CBNAAT or culture-based methods.

Ideally, triage and confirmatory testing should be integrated at the same level of care, especially in high patient-volume settings.^8^ The 2024 WHO target product profiles for rapid TB tests at the peripheral level call for a minimum sensitivity of 90% for sputum-based assays, 80% for non-sputum low-complexity assays, 75% for non-sputum near point-of-care assays, and 65% for non-sputum point-of-care assays, with a minimum specificity of >98%.^7^

Emerging non-sputum-based methods, such as digital chest X-rays combined with telemedicine or computer-aided decision algorithms or those using blood, urine, stool, tongue and oral swab, aerosols and exhaled breath condensate, offer potential as triage and confirmatory tests. These methods are less invasive and more accessible, making them valuable for children who cannot produce sputum and reassuring for parents.^9–14^

This mini-review summarizes the performance of non-sputum-based assays for childhood TB diagnosis and evaluates their potential as triage tools.

## METHODS

**Figure 1.**
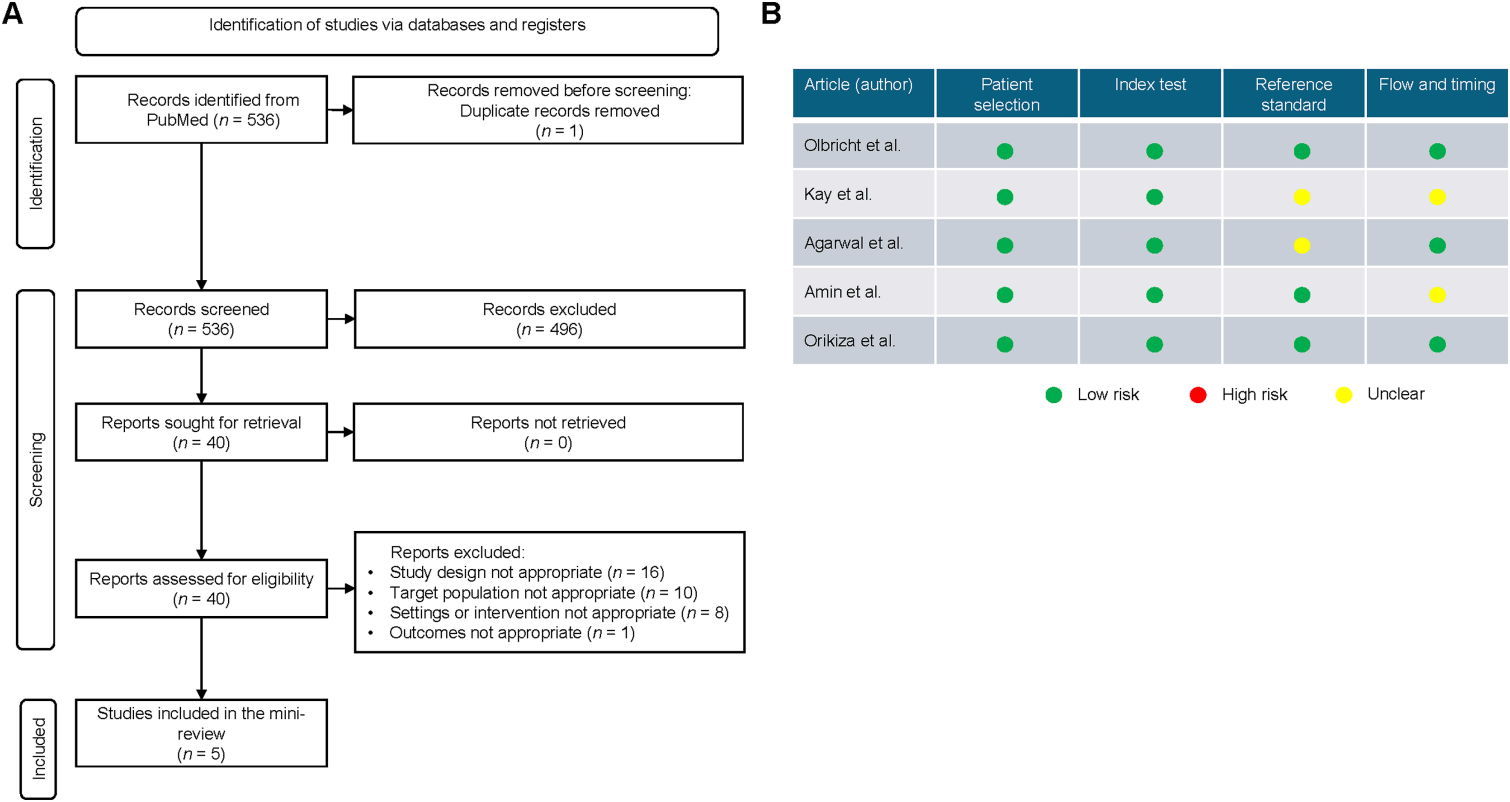
**A)** Mini-review flow diagram according to PRISMA guidelines; **B)** QUADAS-2 risk of bias assessment for included studies. PRISMA = preferred reporting items for systematic reviews and meta-analyses; QUADAS-2 = quality assessment of diagnostic accuracy studies-2.

A search of the PubMed database was conducted to identify relevant articles published between January 2022 and May 2024 on non-sputum-based assays for childhood TB diagnosis according to Preferred Reporting Items for Systematic reviews and Meta-Analyses (PRISMA) guidelines (Figure 1A). This timeframe was based on the last review on the topic and the start of the present mini-review.^14^ PubMed was selected for its extensive coverage of biomedical research. The search strategy employed a combination of keywords and Medical Subject Headings (MeSH) terms related to non-sputum-based TB diagnostics, which are listed in Supplementary Data. The risk of bias in each included study and relevance of primary diagnostic tests was assessed using QUADAS-2 (Figure 1B).^15^

Both the Microbiological Reference Standard (MRS) and the Clinical Reference Standard (CRS) were applied to assess confirmatory and triage test performances.^16,17^ The MRS identified individuals with microbiologically confirmed pulmonary TB (‘Confirmed TB’) through positive sputum culture or *Mycobacterium tuberculosis* DNA detection using CBNAAT like Xpert^®^ MTB/RIF (Cepheid, Sunnyvale, CA, USA) or Xpert^®^ Ultra. The CRS differentiates between microbiologically confirmed TB and clinical TB without microbiological evidence. It also includes chest X-ray evaluation and all subjects receiving WHO-recommended antibiotic therapy. The CRS was selected as it better reflects real-world conditions where diagnostic decisions are based on a combination of clinical, historical, and microbiological evidence. Sensitivity, specificity, negative predictive values (NPV), positive predictive values (PPV), and the overall diagnostic accuracy (e.g., the area under the receiver operating characteristic [ROC] curve; AUC) were recalculated using the MRS or CRS.

The CRS was applied to assess overall performance in evaluating non-sputum confirmatory diagnostics as potential triage tools. Additional criteria were considered to determine whether these assays would meet the latest WHO expectations, including sample collection, cost, practicality, time to results, development phase, maintenance, and energy requirements. These criteria were arbitrarily weighted, considering application in low-income settings as follows: test specificity (weight factor, WF = 5), sample collection (WF = 4), cost (WF = 3), practicality (WF = 3), time to results (WF = 2), development phase (WF = 1), maintenance (WF = 1) and energy requirements (WF = 1).

## RESULTS

**Figure 2.**
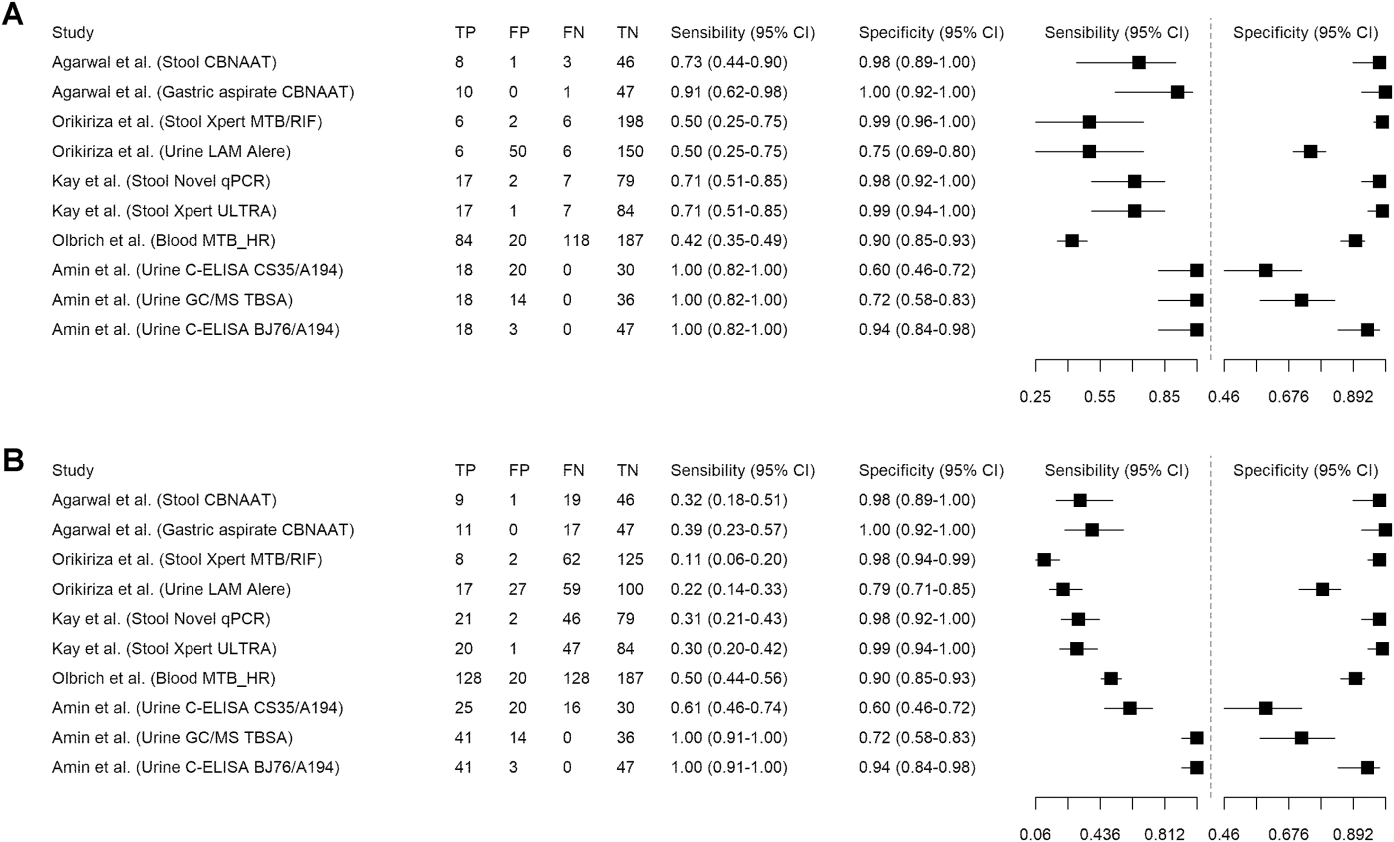
Sensitivity and specificity of confirmatory diagnostic **A)** or triage **B)** tests for TB in children aged <15 years. TP = true-positive; FP = false-positive; FN = false-negative; TN = true-negative; CI = confidence interval; CBNAAT = cartridge based nucleic acid amplification test .

Between January 2022 and May 2024, five studies on non-sputum-based assays for childhood TB diagnosis were included in the present mini-review (Figure 1A and B). The Table and Figure 2A display the confirmatory diagnostic accuracy of these tests using the MRS. For urine samples, LAM detection tests have sensitivities ranging from 0.50 (95% CI 0.25–0.75) for the Determine TB LAM Ag^18^ (Abbott Laboratories, Chicago, IL, USA) test to 1.00 (95% CI 0.82–1.00) for C-ELISA and gas chromatography–mass spectrometry (GC/MS) methods.^19^ Specificity varies from 0.60 (95% CI 0.46–0.72) for the C-ELISA with the CS35/A194 antibody pair to 0.94 (95% CI 0.84–0.98) for the C-ELISA with the BJ76/A194 antibody pair, with the highest diagnostic accuracy of 95.6% achieved by the C-ELISA (BJ76/A194) test.^19^ For stool samples, sensitivity ranges from 0.50 (95% CI 0.25–0.75), for Xpert MTB/RIF,^18^ to 0.73 (95% CI 0.44–0.90), for CBNAAT,^20^ with specificity ranging from 0.98 (0.92–1.00), for the novel quantitative polymerase chain reaction (qPCR),^21^ to 0.99 (95% CI 0.96–1.00), for Xpert MTB/RIF.^18^ The highest diagnostic accuracy of 96.2% is noted with Xpert MTB/RIF.^18^ For blood samples, the MTB-HR test has a sensitivity of 0.42 (95% CI 0.35–0.49) and a specificity of 0.90 (95% CI 0.85–0.93), yielding an overall AUC of 66.3%.^16^

**Table.** Performance of confirmatory diagnostic and triage tests for TB in children aged <15 years.

Study	Agarwal et al.^20^		Orikiriza et al.^18^		Kay et al.^21^		Olbrich et al.^16^	Amin et al.^19^		
Year	2022		2022		2024		2024	2022	
Journal	Indian Journal of Tuberculosis		European Respiratory Journal		Lancet Microbe		Lancet Infectious Diseases	PLoS One		
Country	India		Uganda		Eswatini, Mozambique and Tanzania		South Africa, India, Malawi, Mozambique and Tanzania	Peru		
Age group	6 months–12 years		<2 years		10–19 years		<15 years	1–10 years		
Specimen	Stool	Gastric aspirate	Stool	Urine	Stool		Blood	Urine		
Test evaluated	CBNAAT	CBNAAT	Xpert MTB/RIF	Determine TB LAM Ag	Novel qPCR	Xpert Ultra	MTB-HR TB	C-ELISA (CS35/A194)	GC/MS (TBSA)	C-ELISA (BJ76/A194)
Confirmatory diagnostic*										
Sample size										
Total	58	58	212	212	105	109	409	68	68	68
TB confirmed*	11	11	12	12	24	24	202	18	18	18
No TB^†^	47	47	200	200	81	85	207	50	50	50
PPV	0.89	1.00	0.75	0.11	0.90	0.94	0.81	0.47	0.56	0.86
NPV	0.94	0.98	0.97	0.96	0.92	0.92	0.61	1.00	1.00	1.00
Diagnostic accuracy	0.93	0.98	0.96	0.74	0.91	0.93	0.66	0.71	0.79	0.96
Triage^‡^										
Sample size										
Total	75	75	197	203	148	152	463	91	91	91
TB confirmed*	28	28	70	76	67	67	256	41	41	41
No TB^†^	47	47	127	127	81	85	207	50	50	50
PPV	0.90	1.00	0.80	0.39	0.91	0.95	0.86	0.56	0.75	0.93
NPV	0.71	0.73	0.67	0.63	0.63	0.64	0.59	0.65	1.00	1.00
Diagnostic accuracy (AUC)	0.73	0.77	0.68	0.58	0.68	0.68	0.68	0.60	0.85	0.97

* Using MRS.

† Unlikely TB and/or controls.

‡ Using CRS.

CBNAAT = cartridge-based nucleic acid amplification test; LAM = lipoarabinomannan; qPCR = quantitative polymerase chain reaction; C-ELISA = capture enzyme-linked immunosorbent assay; GC/MS = gas chromatography–mass spectrometry; PPV = positive predictive value; NPV = negative predictive value; AUC = area under the ROC curve; MRS = microbiological reference standard; CRS = clinical reference standard.

Then, using the CRS to evaluate these tests as potential triage tools (Table and Figure 2B), the specificity of urine tests ranges from 0.60 (95% CI 0.46–0.72), using C-ELISA (CS35/A194), to 0.94 (95% CI 0.84–0.98), using C-ELISA (BJ76/A194), with sensitivity from 0.22 (95% CI 0.14–0.33), using Determine TB LAM Ag,^18^ to 1.00 (95% CI 0.91–1.00), using C-ELISA (BJ76/A194) or GC/MS TBSA.^19^ The highest diagnostic accuracy of 96.7% is achieved with the C-ELISA (BJ76/A194).^19^ For stool samples, specificity ranges from 0.98 (95% CI 0.94–0.99), using Novel qPCR, to 0.99 (95% CI 0.94–1.00), using Xpert Ultra,^21^ with sensitivity from 0.11 (95% CI 0.06–0.20), using Xpert MTB/RIF,^18^ to 0.32 (95% CI 0.18–0.51) using CBNAAT,^20^ achieving the highest AUC of 73.3% with CBNAAT. Blood-based MTB-HR reached a specificity of 0.90 (95% CI 0.85–0.93) and sensitivity of 0.50 (95% CI 0.44–0.56), with an overall AUC of 68.0%.^16^

**Figure 3.**
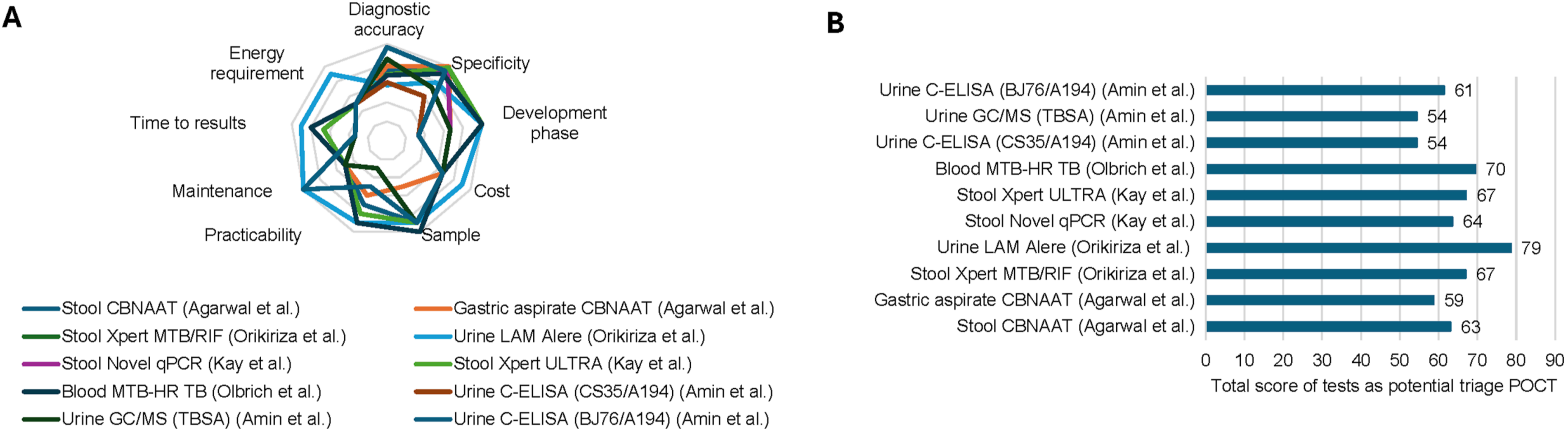
Main features of non-sputum-based tests for the triage of TB in children. **A)** Radar chart showing the main characteristics of the non-sputum-based assays evaluated as rapid triage tools for TB in children. Diagnostic accuracy (WF: 4): high score = high diagnostic accuracy; low score = low diagnostic accuracy; specificity (WF: 5) = high score = high specificity; low score = low specificity; development phase (WF: 1): low score = early (no field evaluation or commercialization); middle score = advanced prototype evaluated as part of a clinical study; high score = assay validated and distributed; cost (WF: 3): low score = >$50; middle score = $7.50–$50; high score = <$7.50; sample (WF: 4): low score = difficult to collect; high score = easy to collect; practicability (WF: 3): low score = trained personnel and laboratory processes required; high score = no trained personnel and laboratory processes required (automated system); maintenance (WF: 1): low score = test or platform requires maintenance, high score = no requirement of maintenance; time to results (WF: 2): low score = >2 h; middle score = <2 h; high score = <30 min; energy requirement (WF: 1): low score = yes, required; high score = no, not required. **B)** Histogram representing the potential of tests evaluated in the review as triage test for childhood TB. CBNAAT = cartridge based nucleic acid amplification test; qPCR = quantitative polymerase chain reaction; GC/MS = gas chromatography–mass spectrometry; POCT = point of care test; C-ELISA = capture enzyme-linked immunosorbent assay; LAM = lipoarabinomannan; WF = weight factor.

Urine-based tests like Determine TB LAM Ag^18^ and blood-based MTB-HR^16^ display several advantages for rapid, community-based triage (Figure 3). Stool-based tests, such as Xpert Ultra, Novel qPCR, CBNAAT, and Xpert MTB/RIF, seem better suited for confirmatory diagnosis. Gastric aspirate-based tests like C-ELISA and GC/MS face challenges in sample collection and point-of-care availability, making them less applicable.

## DISCUSSION

The results reveal significant variability in the accuracy of non-sputum-based assays for diagnosing childhood TB across different studies and settings. Over the past two years, the focus has been on stool, blood, and urine samples, all of which are non-invasive and easier to collect, making them suitable for the rapid detection of *M. tuberculosis* in children.

Regarding confirmatory tests, CBNAAT on gastric aspirate^20^ and the C-ELISA test (BJ76/A194) on urine^19^ are the only assays that meet WHO Target Product Profiles performance criteria for detecting *M. tuberculosis* at the peripheral level, with sensitivity >80% and specificity >98%.^7^ However, gastric aspirate is challenging to collect, limiting its practicality. The C-ELISA test, despite its high sensitivity, requires laboratory processing and varies in effectiveness depending on the antibody pair used, with BJ76/A194 performing better than CS35/A194.^19^ Stool analysis with CBNAAT, despite having lower sensitivity, remains a practical alternative to sputum with a diagnostic accuracy over 90%.

Regarding potential triage tests, the urine Determine TB LAM Ag test^18^ and the blood MTB-HR^16^ test appear to be the best applicable options. The Determine urine test offers a high specificity of 0.79 (95% CI 0.71–0.85), affordability, ease of use, and rapid results within 30 minutes. However, its low sensitivity of 0.22 (95% CI 0.14–0.33) restricts its utility, and its use is limited primarily to individuals with HIV.^22^ Enhancing sensitivity through improved LAM assays using alternative antibody pairs, such as BJ76/A194, seems essential.^19^ The C-ELISA urine test demonstrates high performance, with specificity of 0.94 (95% CI 0.84–0.98) and sensitivity of 1.00 (95% CI 0.91–1.00), but it is less practical for point-of-care settings due to its laboratory-based ELISA format. The MTB-HR^16^ test offers advantages, including its advanced development stage, the use of capillary blood samples, compatibility with Cepheid Xpert devices, and rapid results within 2 h. However, its effectiveness may be influenced by the patient's nutritional status, age, and comorbidities, and its cost-effectiveness as a triage test still requires further evaluation. Emerging blood-based tests, such as the upcoming Q-POC platform^23^ (QuantuMDx Group Ltd, Newcastle upon Tyne, UK)) for the RISK6 assay,^24,25^ could be valuable, delivering results in under 30 minutes from a fingertip sample. Stool analysis using CBNAAT^18,20,21^ remains a viable triage option due to its point-of-care format, cost-effectiveness, practicality, and rapid results (under 2 h) with specificity >95%, although its sensitivity is generally <35%. While CBNAAT on gastric aspirate exhibits the best analytical performance, it is less practical for use in primary care settings.

Several relevant, innovative diagnostic approaches for childhood TB could not be included in this mini-review due to a lack of studies, including the population and raw data necessary to assess test performance against the MRS or CRS. These approaches include digital chest X-ray (coupled with either telemedicine^26^ or artificial intelligence algorithms like CAD4TB^27^), tongue and oral swabs combined with CBNAAT,^28,29^ face masks capturing aerosols combined with molecular detection of *M. tuberculosis*,^30^ and LAM detection in exhaled breath condensate sample.^13^ Nevertheless, the data presented in these studies remain valuable and should be considered for future pediatric research and clinical evaluation. Digital chest X-ray combined with a telemedicine web platform (Biomedical Image Technologies Screen for Pediatric Tuberculosis; BITScreen PTB platform) demonstrated a sensitivity of 16.3–28.2% and a specificity of 91.1%-98.2% for TB identification among children.^26^ Digital chest X-ray combined with CAD4TB (after fine-tuning) achieved an AUC of 0.67 (95% CI 0.53–0.78) using the CRS and 0.78 (95% CI 0.64–0.88) using the MRS, with a sensitivity of 66% and a specificity of 79% (95% CI not reported).^27^ Oral swab specimens (tongue and buccal combined) tested with Xpert Ultra for the diagnosis of pulmonary TB in children displayed a sensitivity of 0.10 (95% CI 0.64–0.16) and specificity of 1.00 (95% CI 0.83–1.0) against CRS, and a sensitivity of 0.25 (95% CI 0.15–0.38) and a specificity of 1.00 (95% CI 0.83–1.0) against the MRS.^29^ In a preliminary proof-of-concept pilot study, the face mask combined with 16S or *rpo*B rRNA detection by reverse transcription polymerase chain reaction (RT-PCR) showed a sensitivity of 96% and a specificity of 85% for TB detection among children aged 2–15 years.^30^ Only one study has provided preliminary data on the potential of LAM detection in exhaled breath condensate as a promising approach for TB diagnosis and treatment monitoring in children.^13^

The strengths of this mini-review include a comprehensive overview of non-sputum-based assays for pediatric TB. However, limitations include heterogeneous study designs (e.g., variability between prospective and frozen sample studies), diverse study populations, lack of raw data needed for performance evaluation using CRS and MRS, and absence of laboratory validation for specificity and cross-reactivity with other mycobacteria.

## CONCLUSION

These findings underscore the need for further evaluation of non-sputum-based assays as triage and confirmatory tests for childhood TB. Integrating these assays into the WHO TB treatment decision algorithms could enhance diagnostic accuracy and treatment, especially for malnourished children, those with HIV, and patients with pneumonia in low-resource settings. Expanded operational research, including cost-effectiveness assessment, would be crucial to improve childhood TB management.

## Supplementary Material


