# Supplementary material for: Non-sputum-based triage and confirmatory diagnostic tests for pediatric TB
Source: IJTLD Open. 2025 Mar 12;2(3):153–9. doi: 10.5588/ijtldopen.24.0484 (PMC11906028; doi:10.5588/ijtldopen.24.0484)
Supplement: Supplementary file 1 [file ijtldopen24-0484_supplementarydata1.pdf]

http://dx.doi.org/10.5588/ijtldopen.24.0484

Non-sputum-based triage and confirmatory diagnostic tests for pediatric TB

Supplementary Data File

Mini-review: non-sputum-based triage and confirmatory diagnostic tests for pediatric tuberculosis

Database: PubMed
Date of Search: November 7, 2024
Results: 536 Records

Search: ("Diagnostic Tests, Routine"[MeSH] OR "Diagnostic Techniques and Procedures"[MeSH] OR "diagnosis"[subheading] OR Diagnostic OR "Diagnostic Test" OR "Diagnostic Techniques" OR Procedures OR "Xpert MTB" OR "RIF assay" OR "MTB Host" OR MTB-HR OR "MTB HR" OR "RIF Ultra" OR "triage tools" OR Triage* OR "non-sputum*" OR "TB diagnostic*" OR "diagnostic tools" OR diagnostic OR "TB case detection")

AND (Urine OR urin* OR "mycobacterial lipoarabinomannan" OR "lateral flow lipoarabinomannan" OR "LAM test" OR "blood"[MeSH] OR "fingerstick blood" OR "aerosols"[MeSH] OR aerosol* OR "breath tests"[MeSH] OR breath* OR "nasopharyngeal aspirate" OR "feces"[MeSH] OR stool OR "skin test" OR "mask" OR "tongue swab" OR “Oral swab” OR “Exhaled breath condensate” OR “digital chest-X ray” OR “Computer-aided decision”)

AND ("Infant"[MeSH] OR Infant* OR infancy OR Newborn* OR Baby* OR Babies OR Neonat* OR Preterm* OR Prematur* OR Postmatur* OR "Child"[MeSH] OR Child* OR Schoolchild* OR "School age" OR Preschool* OR Kid OR Toddler* OR "Adolescent"[MeSH] OR Adoles* OR Teen* OR Boy OR Girl OR "Minors"[MeSH] OR Minors* OR "Puberty"[MeSH] OR Pubert* OR Pubescen* OR Prepubescen* OR "Pediatrics"[MeSH] OR Pediatric* OR "Schools"[MeSH] OR "Nursery school*" OR Kindergar* OR "Primary school*" OR "Secondary school*" OR "Elementary school*") AND ("tuberculosis"[MeSH] OR "Tuberculosis/Diagnosis"[MeSH] OR tuberculo* OR TB OR "Koch* Disease" OR "childhood tuberculosis" OR "pediatric tuberculosis" OR "pediatric TB" OR "childhood TB")

AND Filters: from 2022/1/1 - 2024/5/31
